# Structure and Biological Functions of β-Hairpin Antimicrobial Peptides

**Published:** 2015

**Authors:** P. V. Panteleev, I. A. Bolosov, S. V. Balandin, T. V. Ovchinnikova

**Affiliations:** M.M. Shemyakin and Yu.A. Ovchinnikov Institute of Bioorganic Chemistry of the Russian Academy of Sciences, Miklukho-Maklaya Str., 16/10, Moscow, 117997, Russia

**Keywords:** antimicrobial peptides, innate immunity, β-hairpin structure

## Abstract

Antimicrobial peptides (AMPs) are evolutionarily ancient factors of the innate immune system that serve as a crucial first line of defense for humans, animals, and plants against infection. This review focuses on the structural organization, biosynthesis, and biological functions of AMPs that possess a β-hairpin spatial structure. Representatives of this class of AMPs are among the most active antibiotic molecules of animal origin. Due to their wide spectrum of activity and resistance to internal environmental factors, natural β-hairpin AMPbased compounds might become the most promising drug candidates.

## INTRODUCTION

The innate immune system provides immediate protection for an organism in response to pathogen introduction through a variety of molecular factors that implement the recognizing and effector mechanisms of its function: cell adhesion molecules, pattern recognition (including Toll-like) receptors, scavenger receptors, peptidoglycan recognition proteins, lectins, pentraxins, components of the complement system, LPS-binding protein, lysozyme, lactoferrin, cytokines, chemokines, and many others responsible for the regulation of the initiation and course of protective reactions [1]. Along with the aforementioned protein factors of innate immunity, endogenous antimicrobial peptides (AMPs), produced in vertebrates, invertebrates, plants, fungi and bacteria, play a special role in the protection of an organism against infection. AMPs are mainly synthesized on ribosomes within precursor proteins and might be subjected to post-translational modifications during the maturation process. Mature AMPs contain several to several dozen amino acid residues and usually have basic properties due to their high content of lysine and arginine [2]. Initially, AMPs isolated from insect hemolymph, amphibian skin secretions, and mammalian phagocytes attracted the attention of researchers due to their ability to inhibit the growth of various microorganisms. As novel AMPs began to appear, it became evident that these are universal and evolutionarily ancient elements of the innate immune system. Later, along with facts indicating a direct effector (antibiotic) action, the new ability of most AMPs to play a regulatory (immunomodulatory) role and participate in the functioning of both the innate and acquired immunity has been revealed [3]. In this regard, two terms can be found in the literature: antimicrobial peptides and host defense peptides; the latter is more often applied in relation to the peptides that coordinate immune processes within the host organism.

Acquired immunity appeared during the process of evolution only with the emergence of jawed fish about 500 million years ago. Since invertebrate organisms lack acquired immunity, they can only rely on their innate immune system when coming into contact with pathogens. It is worth noting that the vast majority (98%) of animal species on Earth are invertebrates, with some representatives having a life cycle of more than 100 years [4]. Taking into account the “evolutionary success” of invertebrates, one can speak of the high performance of their immune defense system. In multicellular organisms, AMPs can be distributed systemically, for example, through hemolymph in insects or expressed by immune cells in the blood of vertebrates, or localize in epithelial tissues, which more often come into contact with pathogens (mucous membranes, skin). The wide range of antibiotic characteristics of AMPs, including those directed against resistant strains of pathogens, a relatively low probability to select AMP-resistant infectious agents, and fast and effective destruction of target cells allow one to tap these peptide compounds as a basis for developing a new generation of drugs [5].

**Fig. 1:**
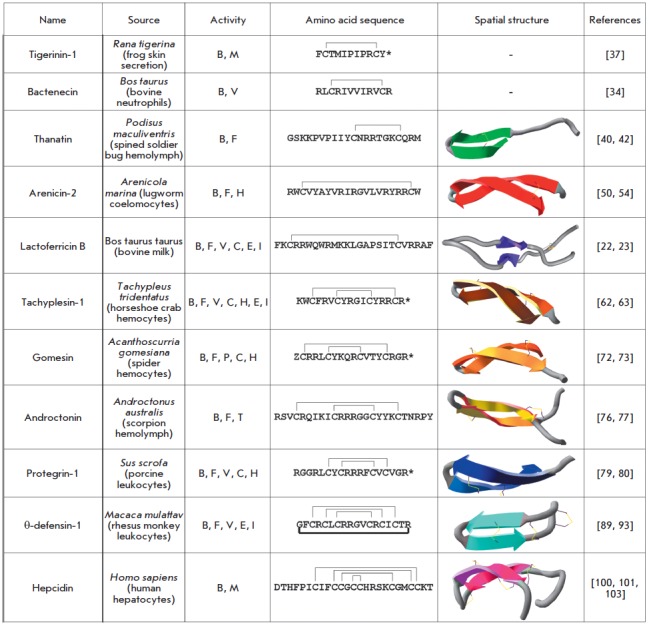
Structure and biological activities of β-hairpin antimicrobial peptides. The disulfide bonds are marked with thin lines. The bold line denotes the peptide bond that forms a θ-defensin cycle. (*) – C-terminal amidation, Z – N-terminal pyroglutamic acid. The biological activities are indicated as follows: B – antibacterial, F – antifungal, V – antiviral, P – antiparasitic, C – anticancer, H – cytotoxic and hemolytic, E – exo- and endotoxin binding, I – immunomodulatory, T – neurotoxic, M – metabolic ones.

About 4,000 natural AMPs have been isolated and characterized thus far [6]. Such physicochemical and biological characteristics as origin, molecular size, primary structure, type of biological activity, mechanism of action, etc. can be used for a classification of AMPs. However, the spatial structure of peptides has turned out to be the most convenient criterion for such classification. The first classification based on the spatial structure was proposed in 1995 [7]. The presence and the number of disulfide bonds in a peptide molecule play a central role in this system. The most widespread classification divides all AMPs into three structural classes. The first class includes peptides that share the α-helical conformation. The second class combines linear peptides that do not form α-helices and can be distinguished by the abundant presence of certain amino acid residues (Gly, Pro, His, Trp). The third class is comprised of peptides that exhibit antiparallel β-strands in their structure. Among the latter group of AMPs are also molecules with a β-sheet structure consisting of three strands (most vertebrate defensins), two strands with a β-hairpin structure, or a mixed structure that includes both β-sheets and α-helices. This review focuses on β-hairpin antimicrobial peptides of animal origin stabilized by disulfide bonds. *Fig. 1* presents data on the multifunctional properties of the main representatives of β-hairpin AMPs, as well as their primary and spatial structures.

The molecular mechanism of the antibiotic action of AMPs in most cases involves a disruption of the cytoplasmic membrane. Three basic models have been proposed to describe the mechanisms of impairment of the barrier function of the cell membrane in the presence of AMPs. The first one, the “barrel-stave” model [8], suggests that AMP molecules, which usually possess a net positive charge, are hydrophobic and amphiphilic in nature, are incorporated into the membrane to form oligomeric ion channels or pores with their inner surface formed by hydrophilic amino acid residues. This model has been proposed particularly for β-hairpin AMP tachyplesin isolated from horseshoe crab hemocytes [9]. Taking into account the high content of basic amino acid residues in the structure of most AMPs, the resulting channels are expected to possess a positively charged inner surface and be anion-selective, which is usually not the case. However, the channels formed by β-hairpin AMP tachyplesin turned out to be anionselective. The second model is based on the description of the toroidal pore formation (the toroidal pore model) and applicable to a wider range of AMPs [10]. The main difference between the abovementioned models is that the second one suggests that the inner hydrophilic surface of the channels includes not only AMP cationic sites, but anionic heads of phospholipids as well. The advantage of this model consists in the higher stability of the complex due to the electrostatic interactions between the AMP and lipids. The third model known as the “carpet model” is based on the detergent-like action of AMPs at high peptide concentrations [11]. The membrane gradually loses its stability as the AMP concentration increases, thus leading to the formation of toroidal gaps and lipid-peptide micelles and finally resulting in cell lysis. The scope of these models for application is conditional, and the final result of AMP action through any of the aforementioned mechanisms is the disruption of the cell membrane barrier function. The selectivity of AMP action is due to the differences in the biochemical composition and electrophysiological properties of the microbial membranes and host cells [12].

Along with the extensive data on the membranotropic properties of AMPs there has been an increasing number of reports on their intracellular targets. In particular, tachyplesin was shown to bind to DNA in the minor groove [13]. When binding to DNA, AMPs can inhibit the replication and transcription processes. Aside from the cytoplasmic membrane and intracellular targets, some AMPs exhibit affinity to the components of bacterial and fungal cell walls. The antibiotic action of such AMPs is thought to be ensured through the inhibition of cell wall biosynthesis. Many AMPs that exhibit antifungal activity (including tachyplesin) are capable of binding to chitin [14].

Besides the inactivation of microorganisms, including bacteria, fungi, protozoa and viruses, AMPs as molecular factors of the innate immune system participate in the regulation of immune reactions. In particular, AMPs possess the ability to opsonize microbes [15]; exhibit chemotactic activity against macrophages, neutrophils, and immature dendritic cells [16]; cause the degranulation of mast cells [17]; modulate dendritic cell differentiation [18]; and they are also involved in the regulation of angiogenesis [19] and possess corticostatic activity [20]. Specific examples of the involvement of β-hairpin AMPs in the regulation of immune reactions are shown below.

Further, we consider the structural and functional characteristics of the main representatives of the β-hairpin AMP family divided into four subgroups, depending on the number of disulfide bonds.

## 1. β-HAIRPIN AMPS STABILIZED BY A SINGLE DISULFIDE BOND

**Lactoferricins**

**Fig. 2:**
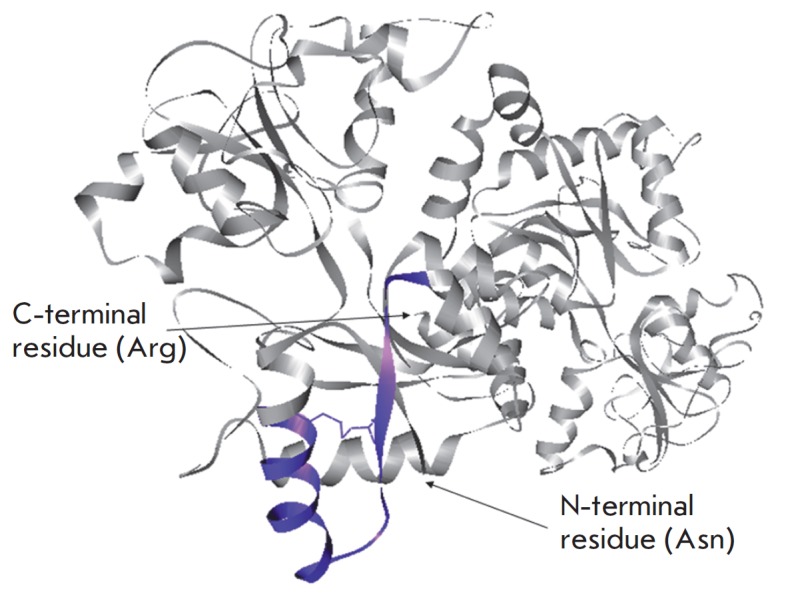
Crystal structure of bovine lactoferrin. The region of the amino acid sequence corresponding to lactoferricin B (residues 17–41) is highlighted in purple

Lactoferricins are the fragments of the functional N-terminal domain of lactoferrin that are produced by limited proteolysis of the protein by pepsin under acidic conditions (*Fig. 2*). Lactoferrin is a multifunctional iron-binding glycoprotein now regarded as one of the essential elements of the defense system against infections in humans and animals. The possible involvement of lactoferrin in resistance against infection was first noticed by Japanese scientists [21]. They isolated two peptides that were the fragments 1–54 and 17–41 of the N-terminal region of bovine lactoferrin and exhibited significantly greater antimicrobial activity than the parent protein. Fragment 17–41, which was later called lactoferricin B [22], is a cationic peptide with a single disulfide bond forming an 18-membered ring between residues Cys2 and Cys20 [23]. Lactoferricin family members have a number of the protective properties intrinsic to lactoferrins isolated from human and bovine milk, with some of these properties being more potent than in the case of the parent protein. Lactoferricins exhibit antimicrobial activity against a broad range of microorganisms, acting both through bactericidal and bacteriostatic mechanisms [24]. The antiviral effect of lactoferricin B is less potent than that of native bovine lactoferrin. Nevertheless, it has an inhibitory effect against a number of viruses [25]. Along with suppression of pathogenic bacteria, lactoferricin B exhibits inhibitory activity against several fungal pathogens, including *Candida albicans *and some dermatophytes [26], *in vitro *antitumor activity against a variety of malignant cell types produced in leukemia, fibrosarcoma and neuroblastoma at concentrations non-toxic to fibroblasts and erythrocytes [27]. It is worth noting that lactoferricin B causes tumor cell death both through necrosis and apoptosis [28, 29]. In addition to that, the peptide exhibits immunomodulatory activity, acting as an anti-inflammatory agent [30]. This effect is explained by the ability of lactoferricin B to bind unmethylated CpG-containing oligonucleotides that are released during bacterial cell death or proliferation and activate inflammatory processes in the organism [31]. Lactoferricin B is also able to actively bind bacterial LPSs, thereby inhibiting the activity of immune system cells [32]. To date, the human lactoferrin-derived fragment hLF1-11, which possesses anti-inflammatory activity, has passed phase I of clinical trials as an immunomodulator [33].

**Bactenecin**

Bactenecin is a small antimicrobial peptide isolated from the neutrophilic granulocytes of cattle that consists of 12 amino acid residues. Cysteine residues at positions 3 and 11 form a disulfide bond resulting in a 9-membered ring [34]. Native bactenecin exhibits a pronounced antibacterial activity against a broad spectrum of both Gram-positive and Gram-negative bacteria, while its hemolytic activity is negligible [35]. A number of bactenecin analogs that have an increased therapeutic index have been obtained. Some of these peptides possess antiviral activity against the herpes virus [36].

**Tigerinin-1**

Tigerinin-1 is a short peptide consisting of 12 amino acid residues. Isolated from the skin of the frog *Rana tigerina*, this peptide is rather different from other amphibian AMPs. The cysteines at positions 2 and 10 form a disulfide bond, which leaves a large portion of the molecule within the 9-membered ring. This structural feature is common to both tigerinin and bactenecin [37]. The similarity is also reflected in the spectra of the peptides’ activity. Tigerinin exhibits antimicrobial activity against a broad range of pathogenic microorganisms [38]. One of tigerinin analogs, tigerinin-1R, is to be mentioned separately, since it is capable of stimulating insulin production. It has been shown that the peptide can cause membrane depolarization and increase intracellular Ca^2+^ concentration in pancreatic β-cells, thus stimulating insulin release. The course of experiments conducted in mice with type II diabetes showed that injection of tigerinin-1R leads to a significant acceleration in glucose decomposition. Furthermore, the peptide does not exert any toxic effect on the organism. The possible development of tigerinin-1R-based drug effective in type II diabetes is currently being discussed [39].

**Thanatin**

**Fig. 3:**
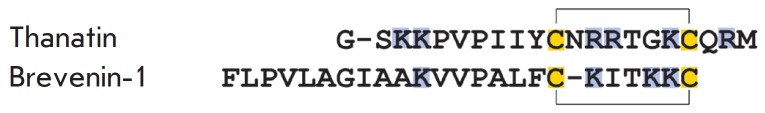
Amino acid sequences of thanatin from P. maculiventris and brevenin-1 from R. brevipoda. Cysteine residues are highlighted in yellow. Basic amino acid residues are highlighted in blue. The disulfide bonds are marked with thin lines

Among the numerous AMPs isolated from insects, thanatin from the spined soldier bug *Podisus maculiventris* is the only peptide molecule with a β-hairpin conformation. Mature thanatin consists of 21 amino acid residues and bears a significant positive charge (+6) at physiological pH [40]. The peptide shares no significant homology with other protective peptides in insects [41]. However, its primary and secondary structures are close to those of the AMPs from the skin secretions of the frog *Rana *[41]. The degree of homology between thanatin and brevenin-1 isolated from the skin of the Japanese frog *R. brevipoda *approaches 50%, with both peptides containing a small loop at the C-terminal part of the molecule, which is formed by a disulfide bond and comprises eight (thanatin) or seven (brevenin) amino acid residues (*Fig. 3*).

The motif typical of brevenins and known as “Rana box” was found in many amphibian AMPs: esculentins, gaegurins, and ranalexins. In all of these molecules, the cycle contains positively charged residues separated by a threonine residue. In thanatin, such a region forms a rigid β-hairpin structure, while the N-terminal fragment of the peptide retains mobility [42].

Thanatin was found to be produced in an insect’s fat body upon experimental infection with pathogenic microorganisms. The peptide is characterized by a wide spectrum of antibacterial and antifungal activities; it is capable of suppressing the growth of Gram-positive and Gram-negative bacteria, as well as filamentous fungi and yeasts at concentrations in most cases not exceeding 10 μM. Furthermore, thanatin shows no hemolytic activity even at concentrations one order of magnitude higher than MIC against bacteria, indicating the high selectivity of its action. Thanatin can inhibit the growth of several multidrug-resistant bacteria, including antibiotic-resistant strains of *Enterobacter aerogenes *and *Klebsiella pneumoniae*. Native thanatin promotes the efficacy of a number of classical antibiotics against clinical isolates expressing the efflux pumps that provide multidrug resistance [43]. In the course of structural and functional studies of thanatin, a series of analogs with improved therapeutic indices were found [44]. A truncated analog of thanatin, R-thanatin, can effectively suppress the growth and formation of biofilms in various MRSA strains both *in vitro *and *in vivo* [45]. Of most interest among the analogs is the more active S-thanatin, wherein the threonin at position 15 has been replaced by serine. This analog has demonstrated high safety and efficacy against a multiresistant strain of *K. pneumoniae *both *in vitro *and in the case of intravenous administration in mice [46, 47]. The ability of thanatin to effectively suppress the growth of fungal pathogens has been applied in the field of plant biotechnology. Thus, transgenic rice and Arabidopsis cultures containing the thanatin gene have demonstrated high resistance to a number of phytopathogens [48, 49].

**Arenicins**

**Fig. 4:**
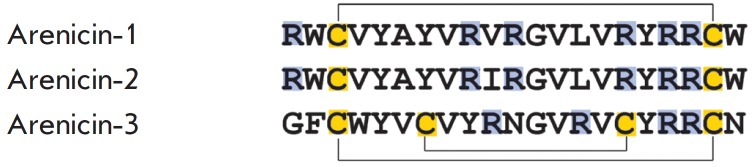
Amino acid structures of arenicin isoforms from A. marina. Cysteine residues are highlighted in yellow. Basic amino acid residues are highlighted in blue. The disulfide bonds are marked with thin lines

Arenicins are cationic peptides isolated from coelomocytes of the lugworm *Arenicola marina *[50]. Arenicin molecules consist of 21 amino acid residues, six of which are positively charged arginine residues, and stabilized with a disulfide bond forming an 18-membered macrocycle (*Fig. 4*). Natural arenicins show high activity against Gram-positive and Gram-negative bacteria, as well as pathogenic fungi and yeasts even under high ionic strength conditions [51]. Studies by a variety of methods have demonstrated the ability of arenicins to disrupt the integrity of bacterial membranes. Obtained experimental data suggest a bactericidal, but not bacteriostatic, mechanism of arenicin action. The study of the antifungal activity of arenicin-1 showed its involvement in the induction of apoptosis [52]. Furthermore, natural isoforms of arenicin exhibit high hemolytic activity. The results of *in vivo *experiments on the assessment of the recombinant arenicin total toxicity have shown that the peptide can be referred to as a Class III toxicity (20 > LD_50_ > 700 mg/kg) for CD-1 mice [53]. The spatial structure of arenicin-2 in aqueous solutions is a twisted β-hairpin stabilized by nine hydrogen bonds and one disulfide bond [54, 55]. When surrounded by a membrane, conformational changes and peptide dimerization take place, leading to the lipid-mediated formation of oligomeric pores [56-58]. A similar mechanism of membrane depolarization resulting in the formation of toroidal pores has been described earlier for β-hairpin AMP protegrin [59].

## 2. β- HAIRPIN AMPS STABILIZED BY TWO DISULFIDE BONDS

**Arenicin-3**

In 2005, the Danish pharmaceutical company Adenium Biotech patented the antimicrobial peptide arenicin-3 isolated from lugworm *A. marina *[60]. The spectrum of its biological activity is similar to the spectra of the earlier discovered arenicin-1 and arenicin-2 [50] (*Fig. 4*). Arenicin-3 differs significantly in structure from the other two members of the family: the homology degrees constitute only 57% and 44% at the nucleotide and amino acid levels of the precursor proteins, respectively. Arenicin-3 consists of 21 amino acid residues, has a net positive charge of +4 and is biologically active at concentrations of less than 1 μM against a broad spectrum of Gram-positive and Gram-negative bacteria, including clinical isolates with multidrug resistance. Unlike arenicin-1 and arenicin-2, this molecule is stabilized by two disulfide bonds and causes almost no lysis of erythrocytes at concentrations of up to 400 μM. High-throughput screening of combinatorial libraries has allowed researchers to create a wide range of arenicin-3 analogs, the structures of which have been patented. The study of the antimicrobial action of arenicin-3 *in vivo *revealed their high therapeutic potential, since the effective doses turned out to be one order of magnitude lower than the maximum-tolerated dose in mouse models of pneumonia and urinary tract infection. One of the arenicin-3 analogs (NZ17074) is currently undergoing preclinical studies as a therapeutic agent against infections caused by multidrug-resistant Gram-negative bacteria [61].

**Tachyplesins and polyphemusins**

Tachyplesins were isolated from the hemocytes of horseshoe crab *Tachypleus tridentatus *[62]. Similar peptides, called polyphemusins, were found in a closely related species: *Limulus polyphemus *[63]. Along with other antimicrobial factors, tachyplesins and polyphemusins are deposited in small-granule hemocytes [64]. Tachyplesins and polyphemusins consist of 17–18 amino acid residues, have a net positive charge of +6 or +7, and are stabilized by two disulfide bonds. Among the notable features of their structure is the presence of an amidated C-terminal arginine residue. Positively charged and hydrophobic residues provide pronounced amphiphilic properties, when in contact with a lipid bilayer [65]. Tachyplesins exhibit marked activity against a broad spectrum of bacteria and yeasts. Polyphemusins show a similar spectrum of antimicrobial activity. However, the MIC values are generally lower, which provides ground for considering the members of this subfamily to be the most active AMPs of animal origin, along with protegrins and arenicins [66]. Moreover, the activity of these peptides is not limited to direct membranotropic action. In addition to the ability to form stable pores and cause depolarization of bacterial membranes, tachyplesin can also bind to intracellular targets, particularly genomic and plasmid DNAs [13]. Moreover, tachyplesin can bind bacterial endotoxins and likewise exhibit immunomodulatory function, participating in the activation of the complement system and regulating the proliferation of cells responsible for the innate immune response [67]. The discovery of polyphemusin antiviral activity against human immunodeficiency (HIV) and influenza viruses led to the development of several therapeutically useful analogs with the appropriate direction of action [68]. Another target for tachyplesins and polyphemusins is tumor cells. Despite the pronounced membranotropic activity, including that in relation to erythrocytes, the antitumor properties of these molecules are associated with such processes as activation of apoptosis [69], inhibition of tumor cell proliferation [70], and activation of the classical complement pathway [71].

**Gomesin**

Gomesin is an AMP isolated from the hemocytes of the spider *Acanthoscurria gomesiana *[72]. The protein is structurally closer to tachyplesins and polyphemusins [73]. The homology level between these AMPs is about 50%. Gomesin contains 18 amino acid residues, including four cysteines that form two disulfide bonds, N-terminal pyroglutamic acid, and a C-terminal amidated arginine residue. Similar modifications of the N- and C-terminal residues are found among peptide hormones. The spectrum of the antimicrobial activity of gomesin is as wide as that of its homologs, and it includes Gram-negative and Gram-positive bacteria, parasitic protozoa, as well as yeast and filamentous fungi. For example, gomesin is capable of binding to the membrane surface and inhibiting the growth of the yeast-like fungus *Cryptococcus neoforma *[74]. Similar to tachyplesins, gomesin exhibits antitumor activity both *in vitro *in relation to melanoma and malignant breast and colon cells, and *in vivo *in melanoma-grafted mice [75]. It is important to note that gomesin has moderate hemolytic activity and toxicity in relation to normal cells.

**Androctonin**

**Fig. 5:**
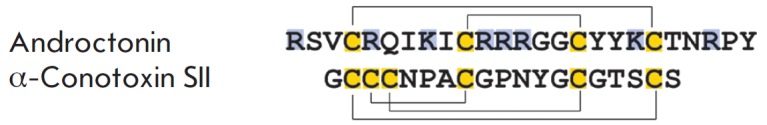
Amino acid sequences of androctonin from A. australis and α-conotoxin SII from C. striatus. Cysteine residues are highlighted in yellow. Basic amino acid residues are highlighted in blue. The disulfide bonds are marked with thin lines

Androctonin is a 25-membered peptide from the hemolymph of the scorpion *Androctonus australis* that contains four cysteine residues forming two disulfide bonds [76]. The synthesis of androctonin occurs constitutively in scorpion hemocytes. An androctonin molecule has a large net positive charge (+8) and contains the RRRGG motif, which is also found in scorpion defensins. The amino acid sequences of androctonins, tachyplesins, and polyphemusins are characterized by a moderate level of homology, but their spatial structures differ in the type of β-turn [77]. In addition, the location of cysteine residues and the position of disulfide bonds in the peptide resemble those of α-conotoxin SII, a blocker of n-acetylcholine receptors isolated from the venom of the marine mollusk *Conus striatus* (*Fig. 5*). Moreover, androctonin was reported to share a comparable with α-conotoxin SII affinity to the nicotinic receptors in *Torpedo* [76], thus suggesting a basis for the development of analgesic drugs.

Androctonin does not cause lysis of mammalian erythrocytes even at high concentrations, up to 150 μM, which may be due to its greater hydrophilicity and mild amphiphilic properties [78]. However, despite the low content (about 30%) of hydrophobic residues as compared with other β-hairpin AMPs, androctonin is able to disrupt the integrity of bacterial membranes. Androctonin is active against Gram-positive and Gram-negative bacteria, yeast and filamentous fungi, while its linear analog, which does not contain any disulfide bonds, exhibits activity only against Grampositive bacteria.

**Protegrins**

The family of protegrins, first isolated from porcine neutrophils more than 20 years ago [79], includes four isoforms consisting of 16–18 amino acid residues. The stability of the protegrin spatial structure is provided by two intramolecular disulfide bonds [80]. Protegrins belong to the family of cathelicidins, AMPs synthesized as the C-terminal region of the precursor protein containing a conserved cathelin domain. Mature protegrins are formed in the extracellular space during proteolytic processing by elastase [81]. As mentioned earlier, protegrins are among the most active AMPs. The MIC of protegrin-1 against the majority of bacterial strains is less than 0.5 μM [82]. For comparison, MSI- 78 is a highly potent analog of one of the best known α-helical AMPs, magainin, which was isolated from the skin of the frog *Xenopus laevis *and acts through a membranotropic mechanism similar to that of protegrins, and exhibits activity against a broad spectrum of bacterial strains at concentrations ~2–4 μM and higher [83]. Aside from its antibacterial action, protegrin can also exhibit activity against yeast and tumor cells [84, 85], as well as viruses [86]. One of the protegrin analogs, the synthetic 17-membered peptide iseganan (IB-367), selected by screening of several hundred analogs with various amino acid substitutions and deletions, should be noted separately [87]. Iseganan exhibits pronounced activity against a broad spectrum of bacteria and fungi, sometimes even exceeding that of natural peptides. The protein preserves its bactericidal activity in a 150 mM NaCl solution, which is equal to the physiological concentration of Na+ in human blood plasma. Iseganan is regarded as a promising agent for treating patients with oral mucositis, patients undergoing anticancer therapy, as well as for treating ventilator-associated pneumonia, cystic fibrosis, and preventing various sexually transmitted diseases [88].

## 3. β-HAIRPIN AMPS STABILIZED BY THREE DISULFIDE BONDS

**Θ-defensins**

**Fig. 6:**
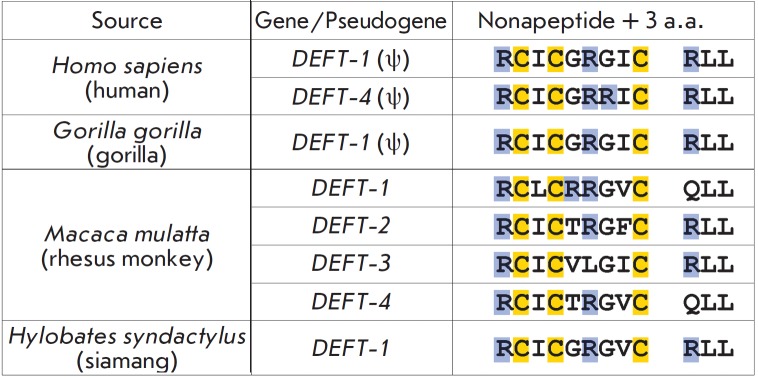
Comparison of primate DEFT genes/pseudogenes expression products [91]. Only the first nine amino acid residues (nonapeptide) in each sequence are incorporated into a mature circular θ-defensin. The other three amino acid residues are eliminated during processing. Cysteine residues are highlighted in yellow. Basic amino acid residues are highlighted in blue

**Fig. 7:**
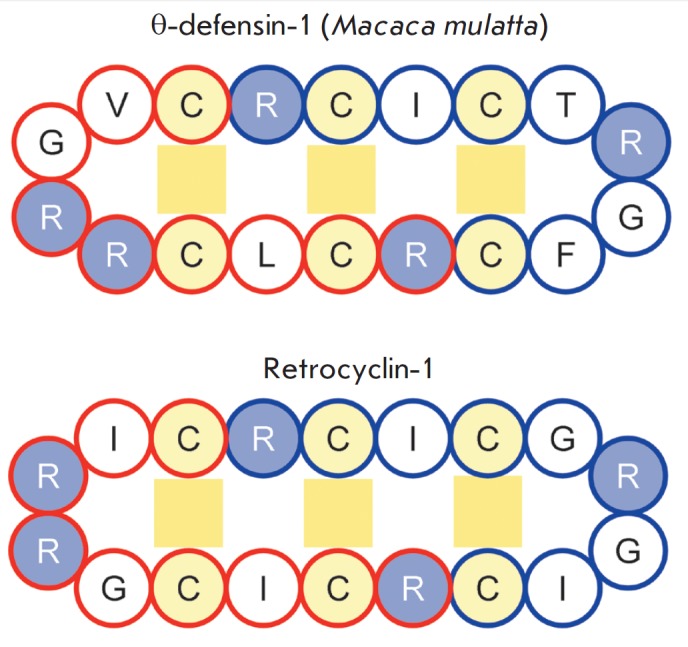
Amino acid sequences of θ-defensin-1 from Macaca mulatta and retrocyclin-1. The amino acid residues of the first and second nonapeptides coupling in the cyclic structure are circled in red and blue, respectively. Cysteine residues are highlighted in yellow. Basic amino acid residues are highlighted in blue

Vertebrate defensins are usually subdivided into three subfamilies: α-, β-, and θ-defensins. All of them share cationic properties, the presence of β-structural regions and six cysteine residues that form three intramolecular disulfide bonds. The subfamilies differ in molecular size, structure and properties, as well as the location of the disulfide bonds. θ-defensins were isolated from the leukocytes of *Catarrhini*, rhesus monkeys and baboons, and are the only example of covalently linked cyclic peptides of animal origin [89, 90]. θ-Defensins have not been found in humans and other most evolutionarily “advanced” primates. It was shown later that human leukocytes produce mRNA encoding precursor proteins of θ-defensins, but the presence of a stop codonin the signal sequence prevents its biosynthesis [91]. Human θ-defensins, known as retrocyclins, have been synthesized using transcript sequence data [92]. Simian θ-defensins are formed by “head-to-tail” splicing of the two nonapeptides, which are the fragments of two independent precursor proteins (*Fig. 6*). Thus, mature θ-defensins consist of 18 amino acid residues and form a β-hairpin structure stabilized by three disulfide bonds [93] (*Fig. 7*). It is worth noting that due to the independent homo- or heterodimeric splicing the number of genes expressing precursor proteins (*DEFT*) defines the finite number of θ-defensin isoforms in a species. Thus, in *Papio anubis *baboon the expression of four* DEFT *genes should theoretically lead to the formation of ten isoforms; however, there were only five peptides found [94]. *DEFT *is a mutated gene of the α-defensin precursor with a stop codon in the region encoding for the mature peptide.

By disrupting the structural integrity of the membrane, θ-defensins and retrocyclins exhibit high antibacterial and antifungal activity at concentrations of about 1 μM. However, unlike the other AMPs described above, they show a one-order decrease in activity following a considerable increase in ionic strength. θ-defensins possess the ability to bind bacterial exotoxins, in particular the anthrax lethal factor from *Bacillus anthracis *[95] and listeriolysin O from *Listeria monocytogenes* [96]. As in androctonins, the spatial structure of θ-defensins is characterized by low amphiphilicity, which is rather unusual for β-hairpin AMPs and results in a low hemolytic activity of the molecules. Due to its low toxicity and the discovery that they exhibit the properties of lectines, θ-defensins are regarded as the prototype of antiviral agents. Numerous studies have demonstrated the ability of retrocyclins to prevent human immunodeficiency [92], influenza [97], and herpes [98] viruses. It is worth noting that the antiviral effect of θ-defensins is not associated with the virotoxic or cytotoxic effect against infected cells. θ-defensins are believed to prevent the spread of enveloped viruses by binding to the surface glycoproteins responsible for the interaction between the virus and the cell during infection. The immunomodulatory activity of θ-defensins, which manifests itself through the ability to inhibit biosynthesis of proinflammatory cytokines, has been demonstrated [99].

## β-HAIRPIN AMPS STABILIZED BY FOUR DISULFIDE BONDS

**Hepcidins**

Hepcidins are a family of β-hairpin AMPs stabilized by four disulfide bonds. Hepcidins are found in many vertebrates at the transcriptome level, but the mature peptides were isolated only from human and fish fluids and tissues [100-102]. Human hepcidin, sometimes referred to as liver-expressed AMP-1 (LEAP-1), was isolated from urine, blood, and the liver. The nucleotide sequence encoding hepcidin is rather conserved between different species, which is especially apparent in mammals. Hepcidins are characterized by the following order of disulfide bonds: Cys1–Cys8, Cys2–Cys7, Cys3–Cys6, Cys4–Cys5, with three of them involved in the interaction of β-strands, whereas the disulfide bridge Cys4-Cys5 causes the deformation typical of molecules of this family in the region of the β-turn and formation of a groove with basic amino acid residues in the inner side and hydrophobic amino acid residues in the outer side [103]. Due to their amphiphilic structure, hepcidins possess a wide spectrum of antimicrobial activity inhibiting the growth of bacteria, filamentous fungi, and yeasts. It is worth noting that mature hepcidins have been detected in fish and isolated from the gills, although the gene is primarily expressed in hepatocytes. Biosynthesis of hepcidin is induced in fish when subjected to pathogenic bacteria. A similar situation was observed in humans: mature peptides were present in urine and blood serum, while the mRNA is predominantly synthesized in the liver.

It has been established that the antimicrobial effect of hepcidin is not due to its direct influence on the bacterial membrane [104], but to its ability to bind nucleic acids [105] and free iron deprivation of the microorganisms [106] necessary for the functioning of superoxide dismutase; i.e. protection against reactive oxygen species. That is why, in spite of the typical properties of AMPs, regulation of the iron metabolism is considered to be its main physiological function in the organism. A series of experiments on knockout mice suggested that hepcidin plays a key role in maintaining iron homeostasis [107]. The lack of hepcidin in the organism leads to metabolic disorders characterized by iron overload. Hepcidin excess is associated with chronic renal failure, anemia, inflammation, and a number of other diseases [108].

## CONCLUSIONS

The data presented above indicate that, despite the relatively small number of known β-hairpin AMPs, their biological functions are very diverse. Summarizing the findings, a conclusion can be drawn that β-hairpin AMPs share a series of essential structural and functional features in terms of the possibility of developing new antibiotics based on their structure, namely: small size (up to 25 amino acid residues), net positive charge and amphiphilic properties sufficient for the manifestation of membranotropic activity against a broad spectrum of bacterial targets, and compact structure stabilized by disulfide bonds providing enhanced proteolytic resistance. The key role of disulfide bonds as a factor that provides the resistance of β-hairpin AMPs to biodegradation has been shown in a number of papers on the example of the analogs of lactoferricin, bactenecin, gomesin, and θ-defensin [109-112]. Thus, all β-hairpin AMPs described in this review share both a similarity in their spatial structures and the ability to effectively destroy target bacterial cells. Their main advantage compared to conventional antibiotics is that bacteria are not yet able to develop effective mechanisms to resist these substances, as this would require significant changes in the structure and electrophysiological properties of the cell membrane [113].

The search for and study of the structural and functional features of β-hairpin AMPs provide exclusively abundant material for developing next-generation drugs. The key objective for researchers laboring on developing new peptide antibiotics is currently the problem of toxicity and increasing the longevity of these molecules in the bloodstream. Due to their structural and functional features, β-hairpin AMPs can be used to develop antibiotics for systemic and surface application, immunomodulators, blockers of exo- and endotoxins, drugs for treating metabolic disorders, anticancer and antiviral drugs, and analgesics. An alternative area of application for β-hairpin AMPs is agricultural biotechnology: namely, the development of transgenic lines of plants that constitutively express AMP genes and, therefore, exhibit high resistance to phytopathogenic microorganisms and other stressful environmental factors.

## Abbreviations

AMP: antimicrobial peptide
LPS: lipopolysaccharide
MIC: minimum inhibitory concentration
HIV: human immunodeficiency virus
LEAP-1: liver-expressed antimicrobial peptide-1 (hepcidin)
MRSA: methicillin-resistant Staphylococcus Aureus
